# Automatic system of diagnosing and treatment in intensive care unit

**DOI:** 10.1186/2197-425X-3-S1-A729

**Published:** 2015-10-01

**Authors:** R Solodova, M Sokolov, V Galatenko, M Petrova

**Affiliations:** Lomonosov Moscow State University, Moscow, Russian Federation; People's Friendship University of Russia, Moscow, Russian Federation

## Introduction

According to statistics, in average 1.7 medical errors occurs daily in the treatment of patients in intensive care [2]. 78% of medical errors are drug dosing errors[3]. Such errors can occur at any stage of the treatment, whether use of the drug, its preparation or administration. Mistakes in medicinal treatment had been done in 187 cases out of 5744 (3.3%) [1]. The greatest number of errors are related to speed of administration (40.1%), a pass-dose, incorrect dosage and delayed use of the drug were, respectively, 14.4%,11.7% and 13.9% [1]. The automatic robotic system is intended to reduce the number of medical errors by automatic dose calculation due to weight, age and etc. It consists of three functional modules: a block of drug administration, patient monitor, the analytical unit (decision support system - processing of received information, the definition of the state, providing recommendations). Data from all devices are automatically directed to the program and program sends commands to pumps. The system is based on national clinical guidelines of patient care in intensive care unit.

## Objectives

The aim of the study was to evaluate the effectiveness of diagnosis and correct treatment sequence of the automatic system.

## Methods

The system performance testing was carried out on patients in intensive care unit. All diagnostic probes (pulse, ECG, blood pressure) of the system was connected to 53 patients on admission. System asks questions step by step and analyze physiological data, make a diagnosis and suggest treatment, after confirmation by the doctor it starts infusions. System corrects velocity of drug administration according to the values of heart rate, saturation, blood pressure.

## Results

In 38 cases system established diagnosis and it was admitted by the doctor, in 2 cases it was not confirmed by the doctor. In 13 cases, the diagnosis of the patient was beyond worked (thromboembolia of pulmonary artery, asthmatic status, cardiopulmonary edema, acute coronary syndrome, hypoglycemic coma, septic and hemorrhagic shock, arrhythmias). The treatment offered by the system was accepted in all diagnosed cases. There were none side effects or unwanted sequels.

## Conclusions

Robotic system helps to diagnosis in 95% of cases and suggest about first therapeutic actions. In time sensitive cases it can prevent misdiagnosis and improper treatment. Further accumulation of material is need to prove reduction in number of medical errors.
